# A case of invasive aspergillosis in CGD patient successfully treated with Amphotericin B and INF-γ

**DOI:** 10.1186/1476-0711-4-4

**Published:** 2005-03-03

**Authors:** Setareh Mamishi, Kamiar Zomorodian, Farshid Saadat, Mohsen Gerami-Shoar, Bita Tarazooie, Seyad Ahmad Siadati

**Affiliations:** 1 Department of Infectious Diseases, Children Medical Center, Tehran University of Medical Sciences, Tehran, Iran; 2 Department of Medical Mycology & Parasitology, School of Public Health and Institute of Public Health Research, Tehran University of Medical Sciences, Tehran, Iran; 3 Department of Immunology, School of Public Health and Institute of Public Health Research, Tehran University of Medical Sciences, Tehran, Iran

## Abstract

**Background:**

Chronic granulomatous disease (CGD) is a rare disorder of phagocytes in which absence of superoxide and hydrogen peroxide production in phagocytes predisposes patients to bacterial and fungal infections. The most common fungal infections in these patients are caused by *Aspergillus *species.

**Case presentation:**

Here, we describe *Aspergillus *osteomyelitis of the ribs and hepatic abscess in a 5-year-old boy. The patient was successfully treated with Amphotericin B and INF-γ.

**Conclusion:**

With respect to the high frequency of aspergillosis in the CGD patient, immune deficiency should be investigated in patients with invasive aspergillosis. Moreover, using antifungal drugs as prophylaxis can improve the quality of life in these patients.

## Background

Chronic granulomatous disease (CGD) is a rare inherited disorder of nicotinamide adenine dinucleotide phosphate (NADPH) oxidase complex of phagocytic cells resulting in failure to generate reactive oxidants and the absence of a respiratory burst [1]. The disease is characterized by recurrent or persistent intra-cellular bacterial and fungal infections. Approximately, the incidence of fungal infections in CGD patients has been reported up to 20% of infections [2]. *Aspergillus spp *are ubiquitous saprophytic fungi and are considered as the major causative fungal agent in these patients [2,3]. The spectrum of infection caused by *Aspergillus *species varies from flu-like pneumonia to life-threatening invasive aspergillosis [4]. The most common form of the aspergillosis in CGD patients is *Aspergillus *pneumonia which can be accompanied by dissemination to the ribs, chest wall and soft tissues [1,2]. Here, we describe a case of invasive aspergillosis in CGD patient with hepatic abscesses and osteomyelitis.

## Case Presentation

A 5-year-old male patient was admitted to Children Medical Center (CMC) with inflammation and swelling in his left mandible and wrist without a history of trauma. In the past, he had suffered from several episodes of pneumonia which started at the age of seven months. On admission, laboratory findings included erythrocyte sedimentation rate (ESR) 84 mm/h, WBC count 12100/mm3 (61% neutrophils, 39% lymphocytes), hemoglobin 11.3 gr/dl and thrombocyte 386000/mm3. As the CRP analysis displayed 20 mg/dl, cephalexin (150 mg/kg/day) was initiated. In his roentgenogram, osteolytic lesions in the distal metaphase of hand and maxillary bone were observed. Considering history of several infections and multifocal osteomyelitis, bone biopsy was performed and his immune system function was evaluated. In the bone biopsy, non-necrotizing granulomatoid lesions were seen. The induration of purified protein derivative reaction was 10 mm diameter. Besides, HIV, hepatitis B surface antigen (HBs), rheumatoid factor and brucella agglutination tests were all negative. The serum IgG level was 1650 mg/dl (normal: 441–1135 mg/dl). IgM and IgA were in high normal range at 250 and 175 mg/dl, respectively. Because no defect was found in his humeral and cellular immunity, the phagocytic cells function was tested with a nitroblue-tetrazolium (NBT) slide test. Based on his hematological and immunological tests (NBT = 0), CGD was considered as underlying disease in this case. Regarding his NBT test, antibiotic therapy was changed from cephalexin to co-trimoxazole (20 mg/kg/day, iv) plus (along with) interferon-γ (50 microgram/m^2 ^every other day). After two weeks of treatment, the patient's condition improved and he was discharged with prescription of both cephalexin (100 mg/kg/day) and co-trimoxazole (10 mg/kg/day) to be taken orally as prophylaxis.

**Figure 1:**
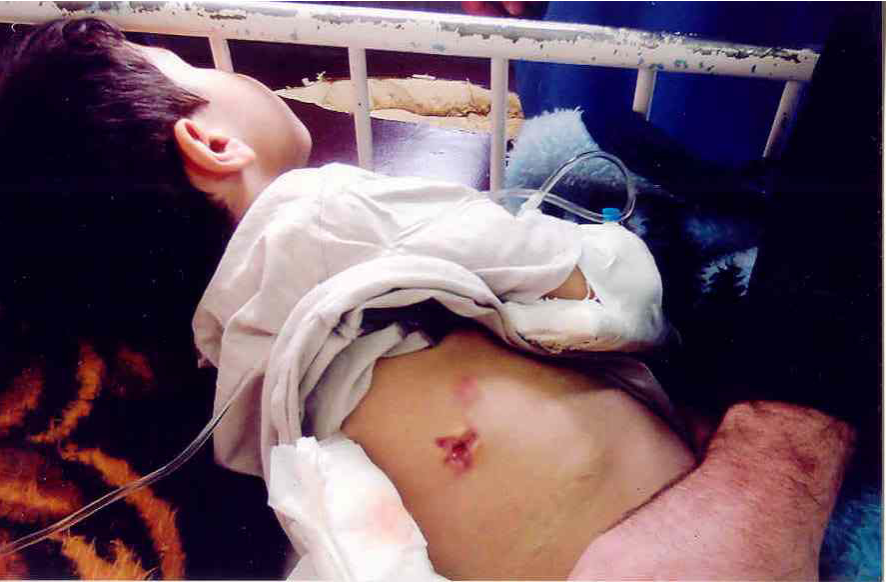
Subcutaneous swelling and granuloma formation in right upper quadrant.

**Figure 2:**
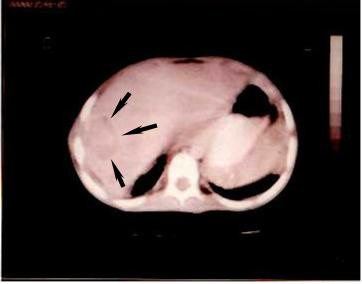
Computerized tomography showed a hypodense area in right lobe of liver with peripheral enhancement

**Figure 3:**
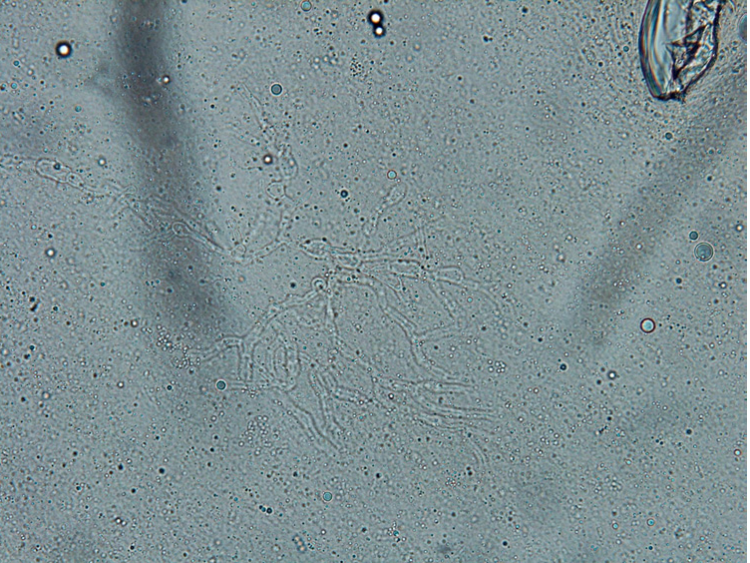
KOH 10% preparation of hepatic abscess showing dichotomous septated hyaline hyphae.

The patient was readmitted to our center after eight months with a tender mass in his right upper quadrant (RUQ) (Fig 1). On admission, his major complaint was severe dyspnea, a persistent cough and also chest and abdominal pain in epigastric area which was started 10 days ago. He was placed on antibiotic therapy including cephalexin (100 mg/kg/day). A computerized tomography (CT) scan of the chest and abdomen was performed which revealed the hypodense area in liver (Fig 2). Adjacent to this opacity, involvement of lower right ribs and reaction to soft tissue were also observed, indicating ribs osteomyelitis. After sonography guided drainage of the above-mentioned hepatic abscess, a sample was sent to the Mycology Department in Tehran University of Medical Sciences. The microscopic examination of clarified specimen with KOH 10% indicated the branched, septated and dichotomous mycelia (Fig. 3). The remaining specimen was also cultured on Brain Heart Infusion agar (BHI), Sabouraud's dextrose agar (S) and Sabouraud's containing 0.005% chloramphenicol (Sc). The S and Sc culture media were incubated at 25°C and BHI at 37°C. The colonies grew rapidly, attaining the diameter of 5 cm within 5 days and their color was bluish green. Cellophane tape preparations and slide cultures demonstrated septated, branched and hyaline hyphae with rough-walled conidiophores and radiated conidial heads. Based on these microscopic and macroscopic findings, *Aspergillus fumigatus *was determined as causative agent in this case. Deoxycholate Amphotericin B (1 mg/kg/day, iv), interferon-γ (50 microgeram/m^2 ^every other day, sc) and rifampicin (10 mg/kg/day) were administered with diagnosis of invasive aspergillosis. The only adverse event observed during treatment was hypokalemia, which was adjusted by administration of potassium chloride 15%. One month after initiation of antifungal therapy, his follow-up CT scan of the abdominal and thoracal region demonstrated relative resolution of hepatic abscess. After four weeks of intravenous treatment, the patient's clinical condition improved. He was discharged upon his parents' responsibility while continuing taking rifampicin (10 mg/kg/day) for two more weeks as a treatment in addition to co-trimoxazole (5 mg/kg/day) and itraconazole (4 mg/kg/day) as long term prophylaxis.

## Discussion

CGD is a rare inherited immune disorder whose prevalence is estimated to be about 1/1,100,000 – 1/1,300,000 individuals worldwide [1]. Similar to the presented case, the most common form of CGD is X-linked recessive that consists of about two thirds of cases and the rest are autosomal recessive [5].

In the absence of minimal oxidative metabolism in CGD which can be ascertained easily using nitro blue tetrazolium (NBT) slide test, other immune mechanisms are triggered [6]. The relative evaluated immunoglobulin levels in the above-mentioned case might be due to persistent antigenic stimulation and it is a common phenomenon in all chronic infections.

This defect is characterized by recurrent or persistent infections due to catalase-positive fungal and bacterial agents despite aggressive antibiotic therapy [1,6]. The incidence of aspergillosis in these patients has been reported to be 78% of all fungal infections [2]. Among *Aspergillus spp*, *Aspergillus fumigatus *is considered to be the predominant cause of invasive aspergillosis in CGD patients [1,7]. Pulmonary aspergillosis has been reported in CGD patients infected with *Aspergillus fumigatus*. As shown in this case, *Aspergillus *might spread from lungs to the bones of thoracic wall and cause osteomyelitis [7-9]. Although *Aspergillus fumigatus *is considered to be the most isolated species, *Aspergillus nidulans *osteomyelitis is reported to have a higher incidence and more mortality rate in these patients [7,9].

The treatment of infections in CGD patients is not easy. Since the underlying immunodeficiency is the most important factor with respect to the outcome of treatment, these patients should be treated either with immunomudulative agents such as recombinant INF-γ or with stimulating factors [10]. Recently, on the basis of cytochrome b (558) expression and NADPH oxidase activity, three different sub-type of X-linked chronic granulomatous disease were described [11]. Therefore, therapeutic response to INF-γ in this case and other X-linked CGD patients might be elucidated. Besides, similar to other systemic fungal infections, antifungal drugs such as amphotericin B should be added to therapeutic regimen of CGD patients with established invasive aspergillosis. Our patient responded to the above-mentioned therapeutic protocol and was discharged with long term anti-microbial and immunomudulatory prophylactic treatment as well as anti fungal drug [12] to enhance the quality of life and lessen the risk of re-infection.
